# PTHR1 Genetic Polymorphisms Are Associated with Osteoporosis among Postmenopausal Arab Women

**DOI:** 10.1155/2021/2993761

**Published:** 2021-12-22

**Authors:** Saba Abdi, Abeer Abdulaziz Almiman, Mohammed Ghouse Ahmed Ansari, Abdullah M. Alnaami, Abdul Khader Mohammed, Naji J. Aljohani, Amal Alenad, Amani Alghamdi, Majed S. Alokail, Nasser M. Al-Daghri

**Affiliations:** ^1^Biochemistry Department, College of Science, King Saud University, Riyadh 11451, Saudi Arabia; ^2^Chair for Biomarkers of Chronic Diseases, Department of Biochemistry, College of Science, King Saud University, Riyadh 11451, Saudi Arabia; ^3^Sharjah Institute for Medical Research, University of Sharjah, Sharjah 27272, UAE; ^4^Obesity, Endocrine, And Metabolic Center, King Fahad Medical City, Riyadh 59046, Saudi Arabia

## Abstract

The parathyroid hormone 1 receptor (PTHR1) plays a crucial role in calcium homeostasis and bone metabolism. However, its genetic role in regulating bone turnover markers (BTMs) in postmenopausal osteoporosis (PMO) remains unclear. Herein, we explored parathyroid hormone (PTH) and PTHR gene variant susceptibility to osteoporosis and their association with various circulating BTM and inflammatory markers in postmenopausal women of Arab ethnicity. In total, 600 postmenopausal Arab women (300-PMO and 300-control) were genotyped for selected SNPs in PTH (rs1459015, rs307253, rs6054, rs307247, rs10500783 and rs10500784), PTHR1 (rs6442037, rs1138518, and rs724449 SNPs) and PTHR2 (rs9288393, rs10497900, and rs897083). Anthropometrics, BTMs, and inflammatory markers were measured. Bone mineral density (BMD) was measured at the lumbar spine L1–L4 and the femoral neck using dual-energy X-ray absorptiometry (DXA). PTHR1 rs1138518 genotype C/T was found to be a significant risk factor for PMO (OR = 1.49, 95% CI 1.0-2.1, *P* = 0.03). The genotypes C/T and T/T of PTHR1 rs1138518 were associated with 25-hydroxy-vitamin D (25(OH)D) regulation. In the PMO group, carriers of the C/T genotype had significantly lower 25(OH)D levels than carriers of the same genotypes in the control group (59.9 (36.7-92.4) nmol/l and 66.4 (43.5-87.8) nmol/l, respectively; *P* = 0.048]. Our study concludes that the PTHR1 rs1138518 genotype could be a potential risk factor for osteoporosis and 25(OH)D regulation in Arab women with PMO.

## 1. Introduction

Osteoporosis is one of the most common chronic conditions affecting the elderly population and a significant public health concern. Worldwide, the age and hormonal changes related to postmenopausal osteoporosis (PMO) affect more than 50% of women aged fifty and above [1–3]. In Saudi Arabia, the prevalence of osteoporosis among individual′s ≥ 50 years is 44.5% in females and 33.2% in males [4–6].

Parathyroid hormone (PTH) plays a significant physiological role in maintaining calcium (Ca^2+)^ and phosphate (P_i_) homeostasis in the body [7]. In response to low circulating Ca^2+^ levels, active PTH directly stimulates the differentiation of bone-forming osteoblasts into bone-resorbing osteoclasts, leading to bone resorption and Ca^2+^ [8]. Two types of receptors for PTH have been recognized. Parathyroid hormone receptor type 1 (PTHR1) is activated by PTH and PTH-related protein (PTHrP). In contrast, parathyroid hormone receptor type 2 (PTHR2) is activated by PTH but not PTHrP [9, 10].

The gene for PTHR1 is located in exon 14 of chromosome 3 and is predominantly expressed in osteocytes and osteoblasts [11]. PTHR1 regulates Ca^2+^ concentration in blood [12], and its abnormal expression corresponds to several metabolic disturbances and critical bone dimorphisms [13].

PTH ligation to PTHR initiates several intracellular signaling pathways [14] that may be G-protein-dependent or independent [15]. A study of PTHR ligands modifications could help understand PTHR function and improve therapeutic options for disorders caused by the defective PTHR1 signaling pathways [9]. Receptor-associated diseases such as hypoparathyroidism and osteoporosis are the most common disorders approved to benefit PTHR1-based therapies [9].

Previously, our group investigated the association between PTH SNPs and the serum levels of 25-hydroxy-vitamin D (25(OH)D) and showed that PTH SNP rs1459015 was associated with higher 25(OH)D levels in several populations [16]. We intend to expand our knowledge of the genetic polymorphisms in PTH and PTHRs among postmenopausal Arab women with osteoporosis from this preliminary evidence. Therefore, this study is aimed at investigating the role of genetic polymorphisms in *PTH* and *PTHRs* in regulating bone turnover markers and inflammation markers in PMO patients. A case-control study was conducted with data from 300 PMO patients and 300 controls of Arab ethnicity. This study was designed to examine the association of PMO with selected SNPs in *PTH* (rs6254, rs1459015, rs307253, rs307247, rs10500783, and rs10500784), *PTHR1* (rs6442037, rs1138518, and rs724449) and *PTHR2* (rs10497900, rs 897083, and rs9288393).

## 2. Materials and Methods

### 2.1. Study Subjects

This case-control study was conducted according to the guidelines of the Declaration of Helsinki and approved by the Ethics Committee of The College of Science, King Saud University, Riyadh, Kingdom of Saudi Arabia (KSA), No. 8/25/36516, and all methods were carried out in accordance with relevant guidelines and regulations. Clinical information of 600 postmenopausal women was collected from the Osteoporosis Registry Database of the Chair for Biomarkers of Chronic Disease (CBCD) at KSU, Riyadh, Saudi Arabia [5, 6, 17]. Participants were recruited from the outpatient clinics of the departments of orthopaedics at King Khalid University Hospital (KKUH), King Fahad Medical City (KFMC), and King Salman Hospital, Riyadh, Saudi Arabia. Written informed consent was obtained from all study subjects prior to their inclusion in the study.

### 2.2. Clinical and Anthropometric Measurements

All information was collected from the registry database. Exclusion criteria were as follows: the presence of any systemic or metabolic disease other than osteoporosis; a food disorder, antacids, steroids, or cortisone; hormone replacement therapy; the use of calcium or a multivitamins supplement; or the use of any drugs that could affect PTH levels. Demographic data included age, sex, and medical history. Anthropometry included height and weight measurements. Body mass index (BMI) was calculated according to the standard equation (kg/m^2^). Bone mineral density (BMD) was measured at the lumbar spine L1–L4 and the femoral neck using dual-energy X-ray absorptiometry (DXA). Diagnosis of osteoporosis (femoral neck *T* − score ≤ −2.5 SD from reference mean of young adult female) was based on the national and regional guidelines for Saudi Arabia and Middle East, respectively [18, 19].

### 2.3. Biochemical Assessment

Fasting blood samples were collected from participants and centrifuged immediately. Serum samples were collected and transported directly to CBCD for storage at -80°C until further analysis. Electrochemiluminescence immunoassays were used to evaluate the levels of total 25(OH)D, N-MID osteocalcin, and *β*-crosslaps (*β*-CTx) using Cobas e411(Roche Diagnostics, Germany). The intra- and interassay CVs were 4.2% and 6% for 25(OH)D, 1.28% and 2.5% for osteocalcin, and 2.4% and 4.2% for the *β*-CTx. Serum intact PTH, osteoprotegerin (OPG), osteopontin (OPN), and receptor activator of nuclear factor-*κ* B ligand (RANKL) levels were measured using multiplex assay kits (Millipore Corporation, Billerica, MA, USA.) as measured previously [20]. The intra- and interassay for intact PTH, OPG, and OPN were 10% and 15%, and for RANKL, 5% and 6%. All samples were measured in duplicates, and the mean was recorded. Interleukin-4 (IL-4) and transforming growth factor *β* (TGF-*β*) were measured using enzyme-linked immunosorbent assay (ELISA) kits (R&D Systems, Minneapolis, MN, USA). The intra- and interassay CVs were 2.4% and 8.2% for IL-4 and 4.7% and 5.2% for TGF-*β*, respectively.

### 2.4. SNPs Analysis

DNA was extracted from whole blood using Prep DNA mini kits (Qiagen, Hilden, Germany) following the manufacturer's instructions. DNA concentration was measured using an ND-1000 Nanodrop spectrophotometer (Thermo Scientific, UK). SNPs from PTH (rs6254, rs1459015, rs307253, rs307247, rs10500783, and rs10500784), PTHR1 (rs6442037, rs1138518, and rs724449) and PTHR2 (rs9288393, rs10497900, and rs897083) were selected for genotyping. Genotyping was performed using TaqMan SNP genotyping assay kits for the 12 candidates. An allelic discrimination real-time PCR was used for SNP evaluation. The target sequence was amplified using 30 ng DNA in 96-well PCR plates and a Bio-Rad CFX96 Real-Time PCR Detection System (Bio-Rad, Milan, Italy). Random samples were genotyped in triplicates for validation. Genotyping cluster plots were reviewed manually, and three SNPs (rs6254, rs10497900, and rs897083) showed poor cluster separation; hence, they were excluded from the data set before further analysis.

### 2.5. Statistical Analysis

Data analysis was carried out using SPSS 21.0 software (IBM, Chicago, IL, USA). Outcome measures were presented as mean ± standard deviation for normal variable and median (1^st^-3^rd^) quartiles for normal and nonnormal variables. Furthermore, analysis of covariance (ANCOVA) was used to compare groups, adjusted for age and BMI. Associations between genotype frequencies in the control and patients' groups were analyzed using Fisher's exact odds ratio (OR) and the corresponding 95% confidence intervals (CIs). Correlation results presented as coefficients and with a *P* value at <0.05 was considered significant.

## 3. Results

The statistical analysis of the clinical characteristics and biochemical indices of subjects is shown in Table 1. A total of 600 postmenopausal women were included in this study based on their osteoporosis status; 300 women with PMO were selected for the PMO group and 300 women without PMO for the control group (CG).

PMO subjects were significantly older and had lower BMI measurements (*P* values < 0.001) than those in the CG. A significant difference was observed in the menopause status between the subjects of the PMO group and the CG (*P* = 0.001). The percentage of women who experienced menopause for 10 or more years was more significant in the PMO group (45.3%) than those in CG (17.7%). The BMD values were measured at the spine, and the femur sites have a high incidence of osteoporosis. BMD values were significantly lower (*P* < 0.001) in the PMO group compared to the CG at all sites evaluated (Table 1).

Among the BTMs, only OPG levels were significantly higher in the PMO group (*P* = 0.037). However, after adjusting values for age and BMI, the results were not significant (Table 1). Among the inflammatory markers, only IL-4 and TGF-*β* levels differed significantly (*P* = 0.01 and *P* = 0.048, respectively) between the PMO group and CG. Although, after adjusting values for age and BMI, no significant difference was detected between circulating levels of IL-4 between the PMO group and CG; however, it was interesting to observe serum TGF-*β* concentrations were significantly lower in the PMO group when compared to the CG (*P* = 0.019) (Table 1).

The genotypic and allelic distributions of the selected SNPs in PMO subjects and control individuals are shown in Table 2. All PTH SNPs selected for this study are located on chromosome 11. No significant difference was observed in genotype or allele frequency distribution of all tested PTH SNPs between the PMO group and CG. The frequencies of the T/T, C/T, and C/C genotypes of PTH rs1459015 were 5.9%, 17%, and 77.1% in the CG and 5.6%, 19.6%, and 74.8% in the PMO group, respectively. The genotype frequencies of PTH rs307253 were as follows: 12.5% in A/A, 38.2% in A/G, and G/G in CG and 14%, 37.1%, and 40.9% in the PMO group. The frequencies of PTH rs307247 genotypes were 10.8%, 35.8%, and 38.5% for A/A, A/G, and G/G in the CG compared to 12.6%, 31.5%, and 38.5%, respectively, in the PMO group. The frequencies of the PTH rs10500783 genotypes were T/T 5.6%, C/T 33.7%, and 61.5% in C/C in CG and T/T 5.9%, C/T 26.6%, and C/C 66.8% in the PMO group. PTH rs10500784 genotype frequencies in the CG were 5.6% for C/C and 33.7% for A/C and 61.5% for A/A compared to 5.2%, 26.2%, and 67.8% for the same genotypes in the PMO group, respectively. Additionally, OR analysis was conducted to evaluate genotypes in the study populations. No statistical correlation was noted between PTH SNP genotypes and the OR adjusted for age and BMI (Table 2).

The distribution frequencies of the PTHR1 SNP genotypes and alleles are also shown in Table 2. All PTHR1 SNPs included in this study are located on chromosome 3. The genotypic distributions of 4 PTHRs SNPs (rs6442037, rs1138518, rs724449, and PTHR2 rs9288393) were found to be as follows. The frequency of PTHR1 rs6442037 G/G was 12.2%, A/G 30.9%, and A/A 50.7% in the CG group and G/G 9.1%, A/G 34.3%, and A/A 46.5% in the PMO group. The statistical distributions of PTHR1 rs1138518 T/T, C/T, and C/C, were 12.8%, 35.4%, and 49.3% in the CG compared to 12.6%, 42%, and 39.2% in the PMO group. PTHR1 rs724449 T/T, C/T, and C/C genotypes frequencies were 25%, 35.1%, and 27.4% in the CG and 24.5%, 41.6%, and 26.2% in the PMO group. Interestingly, PTHR1 rs1138518 heterozygote genotype C/T was significantly associated with the PMO group (*P* = 0.03), where it was at a higher frequency (42%) than in the CG (35.4%) with an OR of 1.49 with a 95% CI (1.04-2.14). No other statistical association was observed between PTHR1 genotypes and the PMO group (Table 2).

The genotypic frequencies of G/G, C/G, and C/C of PTHR2 SNP rs9288393, located on chromosome 2, were 10%, 36.8%, and 53.5% in the CG and 10.5%, 32.2%, and 58% in the PMO group. The allelic frequency distribution of all SNPs was not related to any study groups (Table 2).

Associations between PTHR1 rs1138518 genotypes and circulating levels of BTMs were evaluated using comparative analysis methods (Table 3). PTHR1 rs1138518 homozygote T/T genotype was significantly associated with increased levels (*P* = 0.04) of 25(OH)D in the PMO group. In contrast, heterozygote genotype C/T was significantly associated with reduced levels (*P* = 0.048) of 25(OH)D in the PMO group. No association was observed in either study groups between the T/T or C/T genotype and serum levels of PTH, OPG, OPN, RANKL, osteocalcin and *β*-CTx. Furthermore, the C/C genotype of PTHR1 SNP rs1138518 was not found to be significantly linked to the regulation of any BTMs in this study (Table 3).

Concerning the homozygote T/T genotype, there were no statistically significant differences in serum IL-4, IL-1*β*, leptin, and TGF-*β* levels between the PMO group and the CG (*P* = 0.51, *P* = 0.15, *P* = 0.29, and *P* = 0.55), respectively. Additionally, the C/T genotype was not associated with IL-4, IL-1*β*, leptin, nor with TGF-*β* levels. Inflammatory markers in participants with the C/C genotype did not exhibit any relationship to the PMO group. However, there was a modest reduction in IL-4 levels in the PMO group, which showed a trend towards significance (*P* = 0.064) compared to the CG (Table 4).

## 4. Discussion

The complex polygenic nature of osteoporosis demands the use of multiple genetic analyses to understand outcomes. Therefore, a genetic assessment approach using osteoporosis molecular markers is highly desirable to detect the hormonally related form of the disease. To date, the role of PTH and PTHRs in PMO had not been elucidated. To our knowledge, this is the first study investigating the role of polymorphisms in PTH and PTHRs as key genetic markers for bone turnover regulation in postmenopausal Saudi Arabian women.

The current study identified PTHR1 SNP rs1138518 C/T genotype to be associated with PMO patients. The clinical relevance of PTHR1 rs1138518 in PMO had not been previously reported. To our knowledge, this is the first study to confirm the association between the PTHR1 rs1138518 SNP and PMO. We found a significant difference in the distribution of the rs1138518 genotype among groups. The difference was primarily based on a higher frequency of heterozygote C/T genotype (40.0%) in patients than the control group (34.0%). Our study confirms a previous observation by C. Vilariño-Güell et al. [21], who reported that common variants in PTHR1gene influence BMD in general population. They concluded that the association of PTHR1gene polymorphism on BMD was due to its role in attaining peak bone mass and not due to its effect on bone loss in old age. Similarly, several other previous studies have reported linkage of PTHR1 gene to hip [22] and spine BMD [23]. Additionally, tetranucleotide repeat (AAAG)n polymorphism in the promoter of PTHR1 gene was found to be associated with greater height but not with hip or spine BMD in young adult Japanese women [24]. A study by Zhang et al. failed to find any significant association between BMD and individual SNPs of PTHR1, yet observed an association between haplotype 13 (AATG) and hip peak BMD [25, 26]. Overall, these studies indicate PTHR1 gene variants to influence bone strength and hence osteoporosis risk.

Further, we conducted an association analysis to investigate whether rs1138518 in PTHR1 influenced the circulating levels of selected bone turnover markers. In PMO subjects, while the C/T genotype was found to be linked with a significant reduction in 25(OH) D, the T/T genotype was associated with a significant increase in 25(OH) D levels. This finding suggests that specific genotypes of PTHR1 rs1138518 may play a role in regulating circulating 25(OH) D levels. Thus, carriers of C/T genotype may be at a greater risk if vitamin D deficiency and its related diseases such as PMO.

25(OH)D and PTH are the two foremost regulators of mineral metabolism and are essential for maintaining bone health. These two hormones form a tightly controlled feedback cycle in which the production of 25(OH)D is stimulated by PTH, whereas 25(OH)D exerts negative feedback on PTH secretion [27]. This inverse correlation between 25(OH)D and PTH has been reported in many other studies [28–30].

The PTH-PTHR two-step binding mechanism between PTH and PTHRs helps stabilize the conformational changes implicated by the receptor activation and G-protein [31]. PTHR mediates bone homeostasis and systemic Ca2+ levels. The ligation mainly mediates PTHR activation to endocrine PTH, which triggers internalization and can stimulate sustained endosomal signaling [32, 33]. PTHR can also be activated by the paracrine ligand PTHrP to regulate biochemical processes in bone and other tissues [34].

We acknowledge that there are some limitations to our study. The subject selection involved only postmenopausal Arab women, and therefore, our findings may have limited relevance for different populations. As such, the results could differ using other ethnic groups. Furthermore, the sample size in the present study was small, which could affect applying this finding to a more significant cohort. No association of PTH or PTHR2 polymorphism was noted in the subjects. We also did not measure circulating Ca2+ levels in subjects of this study as a key component in the PTH/25(OH)D feedback loop, and this data could be further investigated. Despite these limitations, to the best of our knowledge, this study is the first to evaluate the relationships between PTHR1 polymorphisms and bone turnover markers in PMO. Unlike earlier SNP-based studies, which mostly focused on the correlation between the genetic polymorphisms of bone regulation and osteoporosis phenotypes, we investigated the possible effects of susceptibility SNPs on serum levels of BTMs.

## 5. Conclusions

Our study is the first to identify that PTHR1 rs1138518 is substantially associated with PMO among Saudi Arabian postmenopausal women. The C/T heterozygote genotype was found to impact the PTH-25(OH)D feedback significantly. The T/T genotype plays a role in maintaining high 25(OH)D levels in PMO patients. Therefore, PTHR1 rs1138518 could represent an integrative biomarker for 25(OH)D and PTH levels in PMO Arab patients. Further molecular functional mechanism studies of the role of PTHR1 rs1138518 in PMO regulation are necessary to clarify this correlation.

## Figures and Tables

**Table 1:** Anthropometric and biochemical characteristics of the subjects according to the study groups.

Parameters	CG N = 300	PMO N = 300	P- value	P- value^∗^
General characteristics				
Age (year)	53.9 ± 6.1	57.8 ± 7.9	<0.001	
BMI (kg/m^2^)	34.3 ± 5.9	31.5 ± 6.3	<0.001	
Menopause status (≥10 years)	50 (17.7)	129 (45.3)	<0.001	0.001

*T*-score and BMD				
*T*-score AP spine #	−0.31 ± 0.83	−2.69 ± 0.67	<0.001	<0.001
*T*-score femur	0.44 ± 1.0	−1.08 ± 0.9	<0.001	<0.001
BMD spine (g/mm^2^)	1.15 ± 0.14	0.85 ± 0.09	<0.001	<0.001
BMD femur (g/mm^2^)	1.03 ± 0.12	0.84 ± 0.12	<0.001	<0.001

Bone turnover markers				
25(OH)D (nmol/l)	61.1 (37.8-84.5)	66.3 (39.1-93.0)	0.20	0.70
PTH (pg/ml)	12.31 (7.59-21.05)	15.41 (7.81-34.98)	0.10	0.40
OPG (pg/ml)	752.1 (523.9-995.3)	853.6 (617.8-1150.7)	0.037	0.43
OPN (ng/ml)	2.32 (1.26-3.36)	2.70 (1.46-4.10)	0.11	0.57
RANKL (pg/ml)	33.18 (18.51-61.02)	28.88 (21.77-48.30)	0.50	0.33
*β*-CTx (ng/ml)	0.07 (0.04-0.11)	0.07 (0.04-0.11)	0.59	0.83
Osteocalcin (ng/ml)	8.89 (3.2-13.13)	9.16 (3.45-14.19)	0.58	0.72
Inflammatory markers				

Interleukins				
IL-4 (pg/ml)	7.04 (3.92-10.33)	4.47 (2.52-9.35)	0.012	0.06
IL-1*β* (pg/ml)	1.72 (0.39-2.72)	1.64 (0.33-2.69)	0.38	0.17

Adipocytokines				
Leptin (ng/ml)	16.14 (7.36-33.1)	20.27 (8.3-34.23)	0.32	0.15

Growth factors				
TGF-*β* (ng/ml)	40.26 (31.47-52.24)	37.58 (18.10-48.56)	0.048	0.019

Note: data presented as mean ± standard deviation for normal variables while the median (quartile 1–quartile 3) presented for nonnormal variables; ^#^nonnormal variables; *P* value < 0.05 considered significant, ^∗^ indicates adjusted *P* value for age and BMI.

**Table 2:** Genotype and allele frequency distribution of PTH and PTHRs SNPs in the study groups.

Gene	SNP ID	Chromosome: position	Gen.	CG	PMO	Adjusted OR (95% CI)	Adjusted *P* value
				*N* (%)	*N* (%)		
PTH	rs1459015	11 : 13500728	T/T	17 (5.9)	16 (5.6)	0.98 (0.48-1.98)	0.9
			C/T	49 (17.0)	56 (19.6)	1.19 (0.77-1.82)	0.43
			^∗^C/C	222 (77.1)	214 (74.8)	1	
			T	83 (14.4)	88 (15.4)	1.08 (0.78-1.49)	0.64
			C	493 (85.6)	484 (84.6)	1	
	rs307253	11 : 13488070	A/A	36 (12.5)	40 (14.0)	1.05 (0.62-1.76)	0.87
			A/G	110 (38.2)	106 (37.1)	0.91 (0.62-1.32)	0.60
			^∗^G/G	110 (38.2)	117 (40.9)	1	
			A	182 (35.5)	186 (35.4)	0.99 (0.77-1.28)	0.95
			G	330 (64.5)	340 (64.6)	1	
	rs307247	11 : 13491931	A/A	31 (10.8)	36 (12.6)	1.17 (0.68-2.1)	0.57
			A/G	103 (35.8)	90 (31.5)	0.88 (0.6-1.3)	0.52
			∗G/G	111 (38.5)	110 (38.5)	1	
			A	165 (33.7)	162 (34.3)	1.03 (0.79–1.34)	0.83
			G	325 (66.3)	310 (65.7)	1	
	rs10500783	11 : 13552067	T/T	16 (5.6)	17 (5.9)	0.98 (0.48-2.0)	0.97
			C/T	97 (33.7)	76 (26.6)	0.73 (0.51-1.04)	0.08
			∗C/C	177 (61.5)	191 (66.8)	1	
			T	129 (22.2)	110 (19.4)	0.84 (0.63–1.12)	0.23
			C	451 (77.8)	458 (80.6)	1	
	rs10500784	11 : 13552278	CC	16 (5.6)	15 (5.2)	0.86 (0.41-1.78)	0.68
			A/C	97 (33.7)	75 (26.2)	0.71 (0.49-1.01)	0.06
			^∗^AA	177 (61.5)	194 (67.8)	1	
			A	129 (22.2)	105 (18.5)	0.79 (0.59–1.06)	0.11
			C	451 (77.8)	463 (81.5)	1	

PTHR1	rs6442037	3 : 46888056	G/G	35 (12.2)	26 (9.1)	0.82 (0.47-1.43)	0.48
			A/G	89 (30.9)	98 (34.3)	1.21 (0.83-1.75)	0.32
			∗A/A	146 (50.7)	133 (46.5)	1	
			G	159 (29.4)	150 (29.2)	0.99 (0.76–1.29)	0.93
			A	381 (70.6)	364 (70.8)	1	
	rs1138518	3 : 46902784	T/T	37 (12.8)	36 (12.6)	1.23 (0.73-2.10)	0.43
			C/T	102 (35.4)	120 (42.0)	1.49 (1.04-2.14)	0.03
			^∗^C/C	142 (49.3)	112 (39.2)	1	
			T	176 (31.3)	192 (35.8)	1.22 (0.95–1.57)	0.11
			C	386 (68.7)	344 (64.2)	1	
	rs724449	3 : 46894191	T/T	72 (25.0)	70 (24.5)	1.02 (0.65-1.62)	0.92
			C/T	101 (35.1)	119 (41.6)	1.24 (0.82-1.87)	0.30
			^∗^C/C	79 (27.4)	75 (26.2)	1	
			T	245 (48.6)	259 (49.1)	1.02 (0.80–1.30)	0.89
			C	259 (51.4)	269 (50.9)	1	

PTHR2	rs9288393	2 : 208404685	G/G	29 (10.1)	30 (10.5)	0.96 (0.55-1.67)	0.88
			C/G	106 (36.8)	92 (32.2)	0.81 (0.56-1.15)	0.23
			^∗^C/C	154 (53.5)	166 (58.0)	1	
			G	164 (28.4)	152 (26.4)	0.91 (0.70–1.17)	0.45
			C	414 (71.6)	424 (73.6)	1	

Note: data presented *N* (%). *P* value significant at 0.05 and 0.01 level using multinomial logistic regression and chi-square test. Odds ratio (OR) and *P* value adjusted for adjustment of multiple comparisons. Significant values are in bold text. ^∗^Reference.

**Table 3:** Comparison of BTMs levels according to PTHR1 rs1138518 genotypes in the study groups.

Parameters	PTHR1 rs1138518		*P* values	*P* values^∗^
	CG	PMO		
Genotype T/T				
25(OH)D (nmol/l)	58.2 (39.6-81.0)	81.2 (60.8-101.7)	0.02	0.04
PTH (pg/ml)	12.1 (6.5-26.1)	15.2 (6.7-48.1)	0.67	0.74
OPG (pg/ml)	866.3 (621.2-1081.7)	744.6 (558.4-979.4)	0.36	0.65
OPN (pg/ml)	2505.2 (1334.6-3121.7)	3181.5 (1825.4-4863.1)	0.18	0.28
RANKL (pg/ml)	40.6 (23.3-68.3)	32.8 (25.4-77.6)	0.96	0.96
*β*-CTx (ng/ml)	0.1 (0.0-0.1)	0.1 (0.0-0.1)	0.46	0.58
Osteocalcin (ng/ml)	5.8 (2.9-14.5)	7.1 (1.8-13.5)	0.036	0.09

Genotype C/T				
25(OH)D (nmol/l)	66.4 (43.5-87.8)	59.9 (36.7-92.4)	0.51	0.048
PTH (pg/ml)	12.2 (7.9-17.8)	17.8 (10.6-69.6)	0.016	0.10
OPG (pg/ml)	719.8 (471.5-887.6)	854.9 (609.3-1056.2)	0.07	0.54
OPN (pg/ml)	2298.0 (1397.7-2952.2)	2651.6 (1706.7-3986.1)	0.12	0.66
RANKL (pg/ml)	32.0 (13.4-65.5)	29.0 (21.6-48.2)	0.84	0.87
*β*-CTx (ng/ml)	0.1 (0.0-0.1)	0.1 (0.1-0.2)	0.70	0.80
Osteocalcin (ng/ml)	8.6 (3.2-12.5)	10.7 (4.8-15.3)	0.023	0.23

Genotype C/C				
25(OH)D (nmol/l)	60.6 (36.6-82.6)	65.9 (41.8-87.1)	0.24	0.52
PTH (pg/ml)	12.3 (8.4-26.7)	13.6 (6.9-27.5)	0.738	0.55
OPG (pg/ml)	752.1 (513.6-1168.1)	826.7 (641.3-1227.7)	0.252	0.44
OPN (pg/ml)	2356.5 (804.5-3482.8)	1812.1 (689.1-3424.8)	0.458	0.67
RANKL (pg/ml)	33.9 (18.4-62.2)	27.9 (21.2-48.2)	0.535	0.40
*β*-CTx (ng/ml)	0.1 (0.0-0.1)	0.1 (0.0-0.1)	0.690	0.48
Osteocalcin (ng/ml)	9.4 (3.4-13.6)	8.0 (3.5-12.9)	0.699	0.75

Note: data presented as mean ± SD for normal variables while median (Q1–Q3) for nonnormal variables; *P* < 0.05 considered significant. ^∗^Adjusted *P* value for adjustment for multiple comparisons.

**Table 4:** Comparative analysis of inflammatory marker levels according to PTHR1 rs1138518 genotypes in the study groups.

Parameters	PTHR1 rs1138518		*P* values	*P* values^∗^
	CG	PMO		
Genotype T/T				
IL-4 (pg/ml)	5.4 (3.5-7.7)	3.8 (3.0-7.4)	0.44	0.51
IL-1*β* (pg/ml)	1.6 (0.3-2.7)	0.4 (0.3-1.6)	0.16	0.15
Leptin (pg/ml)	22697.3 (11742.5-42916.3)	28803.4 (12919.2-37878.6)	0.84	0.29
TGF-*β* (pg/ml)	40749.5 (37204.7-48681.4)	43097.6 (40137.4-51499.3)	0.62	0.55

Genotype C/T				
IL-4 (pg/ml)	8.2 (4.2-10.6)	4.1 (2.5-9.0)	0.10	0.26
IL-1*β* (pg/ml)	1.8 (0.5-2.7)	2.0 (0.3-2.8)	0.69	0.93
Leptin (pg/ml)	14532.2 (4761.6-27012.4)	20484.0 (10396.4-29426.2)	0.14	0.27
TGF-*β* (pg/ml)	37710.2 (29447.5-52241.4)	34811.5 (17464.4-46481.6)	0.31	0.13

Genotype C/C				
IL-4 (pg/ml)	7.1 (3.7-10.7)	4.6 (2.5-9.3)	0.6	0.06
IL-1*β* (pg/ml)	1.8 (0.4-2.9)	1.6 (0.3-2.7)	0.22	0.09
Leptin (pg/ml)	14405.6 (1020.8-33059.9)	18808.1 (3756.0-31273.4)	0.71	0.39
TGF-*β* (pg/ml)	42041.5 (26232.0-54136.5)	33770.1 (15509.4-50533.2)	0.08	0.09

Note: data presented as mean ± SD for normal variables while median (Q1–Q3) for nonnormal variables; *P* < 0.05 considered significant. ^∗^Adjusted *P* value for adjustment for multiple comparisons.

## Data Availability

The data is available from the corresponding author on reasonable request.
